# Genome-Wide Functional Analysis of the Cotton Transcriptome by Creating an Integrated EST Database

**DOI:** 10.1371/journal.pone.0026980

**Published:** 2011-11-08

**Authors:** Fuliang Xie, Guiling Sun, John W. Stiller, Baohong Zhang

**Affiliations:** Department of Biology, East Carolina University, Greenville, North Carolina, United States of America; The Centre for Research and Technology, Hellas, Greece

## Abstract

A total of 28,432 unique contigs (25,371 in consensus contigs and 3,061 as singletons) were assembled from all 268,786 cotton ESTs currently available. Several *in silico* approaches [comparative genomics, Blast, Gene Ontology (GO) analysis, and pathway enrichment by Kyoto Encyclopedia of Genes and Genomes (KEGG)] were employed to investigate global functions of the cotton transcriptome. Cotton EST contigs were clustered into 5,461 groups with a maximum cluster size of 196 members. A total of 27,956 indel mutants and 149,616 single nucleotide polymorphisms (SNPs) were identified from consensus contigs. Interestingly, many contigs with significantly high frequencies of indels or SNPs encode transcription factors and protein kinases. In a comparison with six model plant species, cotton ESTs show the highest overall similarity to grape. A total of 87 cotton miRNAs were identified; 59 of these have not been reported previously from experimental or bioinformatics investigations. We also predicted 3,260 genes as miRNAs targets, which are associated with multiple biological functions, including stress response, metabolism, hormone signal transduction and fiber development. We identified 151 and 4,214 EST-simple sequence repeats (SSRs) from contigs and raw ESTs respectively. To make these data widely available, and to facilitate access to EST-related genetic information, we integrated our results into a comprehensive, fully downloadable web-based cotton EST database (www.leonxie.com).

## Introduction

Cotton is among most important crops for natural textile fiber oilseed and is planted widely in 70 developed and developing countries, including the U.S., China, India, and Australia [1], [2]. Although there are more than 50 species in the genus *Gossypium*, only four of them are cultivated; these are upland cotton (*Gossypium hirsutum L.*), sea-island cotton (*Gossypium barbadense*), Asian cotton (*Gossypium arboreum*), and Arabian cotton (*Gossypium herbaceum*). Upland cotton is, by far, the most widely planted, accounting for more than 95% of the annual cotton crop worldwide.

There are approximately 45 diploid (2n = 2x = 26) and five tetraploid (2n = 4x = 52) *Gossypium* species. Upland cotton has a complex allotetraploid genome (AADD, 2n = 4x = 52) [3], with a haploid genome size estimated to be around 2.5 Gb [4]. Decoding the cotton genome is a crucial foundation for enhancing research on fiber development, quality, yield, and other important agronomic traits. Although some progress has been made on cotton genetics and agronomic improvement, sequencing of the complete cotton genome is still ongoing, largely because of its overall genetic and structural complexity [3].

Currently, there are several types of cotton genomic resources available, including bacterial artificial chromosomes (BACs), expressed sequence tags (ESTs), linkage maps, and integrated genetic and physical maps [3]. To date, a total of 268,786 ESTs have been deposited in the public database GenBank. This large number of ESTs provides at least three obvious advantages: 1) broad EST coverage is a key landmark for future genome analysis and assembly [5]; 2) ESTs can contribute to more efficient gene discovery and identification, especially from species with unavailable genome sequences [6]; 3) ESTs provide information about gene expression, including tissue- and developmentally specific differences, as well as temporal responses to environmental changes [2]. Udall and co-workers previously assembled cotton ESTs using a total of 185,198 sequence reads from 30 cDNA libraries [7]; however, it now is necessary to re-assemble cotton ESTs because there currently are 268,786 EST reads available. Furthermore, careful investigation of the likely functions of these assembled ESTs will be more important for enhancing cotton molecular genetics, for example, identifying useful new genetic markers.

One example of such genetic markers is simple sequence repeats (SSRs), also termed microsatellites, which are tandem repeats of two-to-six base-pair nucleotide motifs. They vary in length among different genotypes and offer a rich source of allelic polymorphisms. In contrast, SSR flanking sequences are often relatively conserved among genomes, making it possible to develop genetic markers for molecular breeding selection and genotype identification [8]–[10]. Compared with other types of molecular markers, SSRs have a number of advantages including co-dominant inheritance, high abundance, a generally random distribution across the genome, high information content, and reproducibility [9]. There are two classes of SSRs, those located in non-coding genomic regions and those found in ESTs. EST-SSRs generally are more conserved within and across related species and show higher transferability because more variable intron or intergenic sequences are absent from ESTs [11]. Additionally, it is more likely that EST-SSRs are tightly linked to specific gene functions and perhaps some even play a direct role in controlling important agronomic traits [12]. Therefore, EST-SSRs are good tools to facilitate marker-assisted selection (MAS) for breeding. To date, EST-SSRs have been used to screen cotton fiber-related loci from EST libraries generated from the cultivated diploid species *Gossypium arboreum L. cv* AKA8401 [13].

Although it is possible to find polymorphic loci using EST-SSR markers, alone they are not sufficient for uncovering the underlying genetics of highly complex traits, such as disease resistance, yield, and quality, because of their low density of coverage across the genome. Furthermore, there are limited polymorphic SSR markers available to help in discriminating between closely related species [14]. Single nucleotide polymorphisms (SNPs) are the most abundant type of DNA polymorphism in genomes. SNPs are alternative nucleotides present at a given, defined genetic location at a frequency exceeding 1% in a given population. Theoretically, each SNP can have four alleles, but bi-allelic variation has been shown to be the most frequent [15]. SNPs are considered to be the major genetic source of phenotypic variability that differentiates individuals within given species [16]. They have been applied extensively to genome-wide association studies (GWAS) of complex traits [16], fine mapping of QTLs [17], and linkage disequilibrium-based association mapping [18]. Because ESTs are rich in current public databases, it is possible for EST-derived SNPs to be a low-cost and efficient resource for investigating genome-level variability before a draft cotton genome becomes available [14], [19].

MicroRNAs (miRNAs) are short non-coding RNA molecules that regulate protein-encoding gene expression at post-transcriptional levels. The main mechanisms of miRNA action are 1) promoting degradation and 2) inhibiting translation of their target mRNAs [20]. Recently, several investigations have shown that translational inhibition is widespread in the plant kingdom [20], [21]. In plants, primary miRNAs (pri-miRNA) are transcribed by RNA polymerase II from intergenic or intron regions and then folded into pre-miRNA hairpins. DICER-LIKE 1 (DCL1) directs conversion of pri-miRNAs to pre-miRNAs, and their processing into mature miRNAs. These steps mostly are carried out in the nucleus. Mature miRNA duplexes are stabilized by the S-adenosyl methionine-dependent methyltransferase Hua Enhancer 1 (HEN1) and are exported to the cytoplasm with the assistance of the plant homolog of exportin-5, HASTY [22]. Mature miRNAs are generated by unbinding mature miRNA duplexes and then are loaded into the miRNA-induced silencing complex (miRISC). Integrated miRISC acts on a target message by perfect or near-perfect complementary base-pairing [22]. In both plants and animals, many miRNA families are highly conserved through hundreds of million of years of evolution [20]. To date, miRNAs have been identified successfully from plant EST and GSS databases based on sequence conservation and characteristic miRNA features [2], [23], [24]. EST databases also provide evidence on temporal and developmental patterns of miRNA expression. ESTs are considered to be a reliable data source for prediction of miRNAs as well their targets, especially in those species without complete genome information [2], [23], [24].

In this study, we performed global assembly of cotton ESTs available from NCBI, and functional annotation using BLASTx, BLASTn, Gene Ontology (GO), and Kyoto Encyclopedia of Genes and Genomes (KEGG) resources. Using the contigs obtained, we also performed EST-based investigations of comparative transcriptome similarity between cotton and other plant species, sequence polymorphisms, expressed miRNAs and their targets, and SSR analysis. Finally, we integrated these analytical data into a comprehensive web-based database so that EST-related information can be shared and queried publically.

## Results and Discussion

### EST assembly

**Figure 1:**
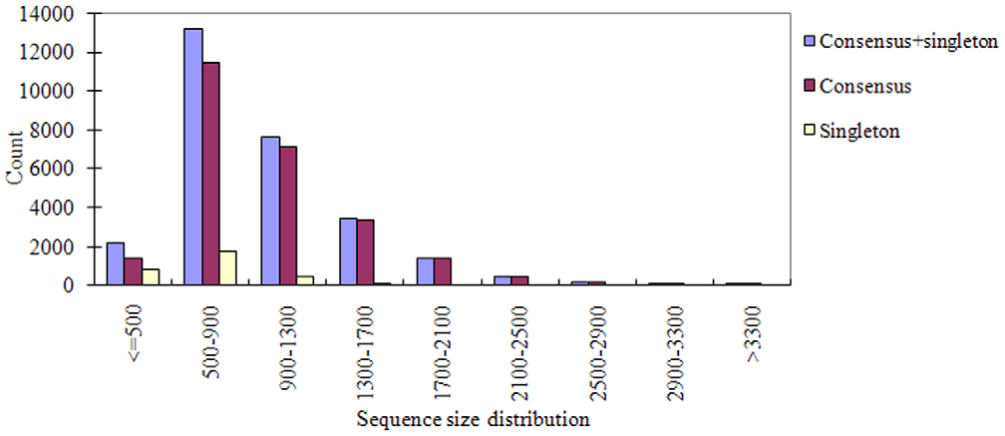
Sequence size distribution of consensus contigs and singletons in cotton.

A total of 268,786 cotton ESTs were collected from NCBI; they have been obtained from different tissues, including fiber, ovule, anther, boll, callus, cotyledon, embryo, leaf, root, stem, seedling, and cultured cells (Table 1). The largest fraction of cotton ESTs is from fiber, with 114,167 sequences or 42.48% of all ESTs available. These ESTs were isolated from different treatments, including cold, cycloheximide, drought, aging, and *Fusariumoxysporum f. sp. vasinfectum* and *Xanthomonascampestris pv. Malvacearum* infections. After pre-processing raw sequences, a total of 235,328 clean ESTs were assembled into 28,432 unique genes (contigs) including 25,371 consensus contigs and 3,061 singletons. Contig lengths ranged from 101 to 4,080 nt (Figure 1). Consensus assemblies shared a similar sequence size distribution with singletons, except that few of the latter were found among longer length contigs. Most assembled contigs fell in the ranges from 500 nt to 900 nt (46.44%) or 900 nt to 1300 nt (26.76%) in length (Figure 1).

**Table 1:** Distribution of sources of raw cotton ESTs from different tissues.

EST library	Count of EST
Anther	51
Boll	5,387
Callus	242
Cell	4
Cotyledon	2,444
Embryo	509
Fiber	114,167
Fiber/Embryo	113
Fiber/Ovule	16,861
Leaf	6,675
Meristematic	44,615
Ovule	53,499
Protoplast	210
Root	6,003
Hypocotyl tissues	1,014
Seedling	2,468
Stem	14,482
Other	42

### Annotation

Because a complete cotton genome is unavailable, it is difficult to determine precise CDS and protein sequences. Gene functions were annotated in two ways: BLASTx against all plant reference proteins data and BLASTn against all plant reference nucleotide data Most ESTs were inferred to be homologous with at least one protein-coding gene counterpart in another plant species, including *Arabidopsis*, rice, maize or grape. However, 6,441 sequences (22.64% of assembled EST contigs and singletons) by BLASTx and 7,992 contigs by BLASTn (Table 2). In total, 4,043 contigs (14.22%) could not be annotated through BLAST searches. In addition, more than 60% of ESTs shared the same or similar annotation amongst BLASTx and BLASTn search results.

**Table 2:** Coding and non-coding contigs inferred by BLASTx and BLASTn.

Method	Coding count	Coding %	Non-coding Count	Non-coding %
BLASTx	21,991	77.35	6,441	22.65
BLASTn	20,510	72.14	7,922	27.86
Common	16,124	56.71	4,043	14.22

**Figure 2:**
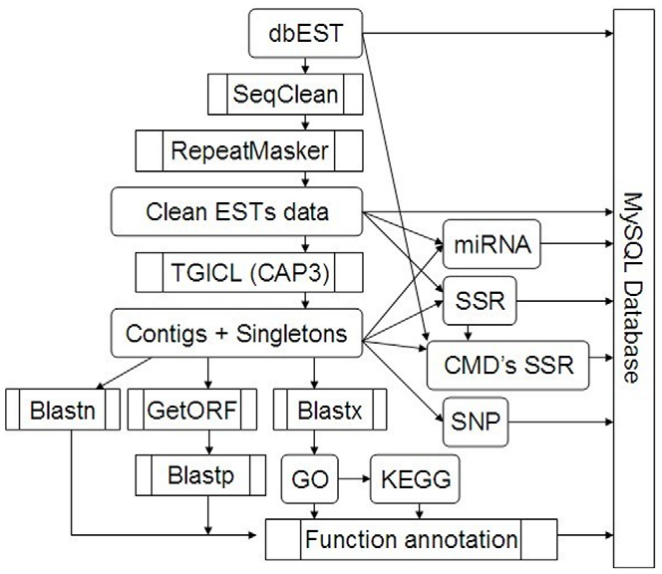
Schematic pipeline for cotton EST assembly, data analysis and database development.

**Figure 3:**
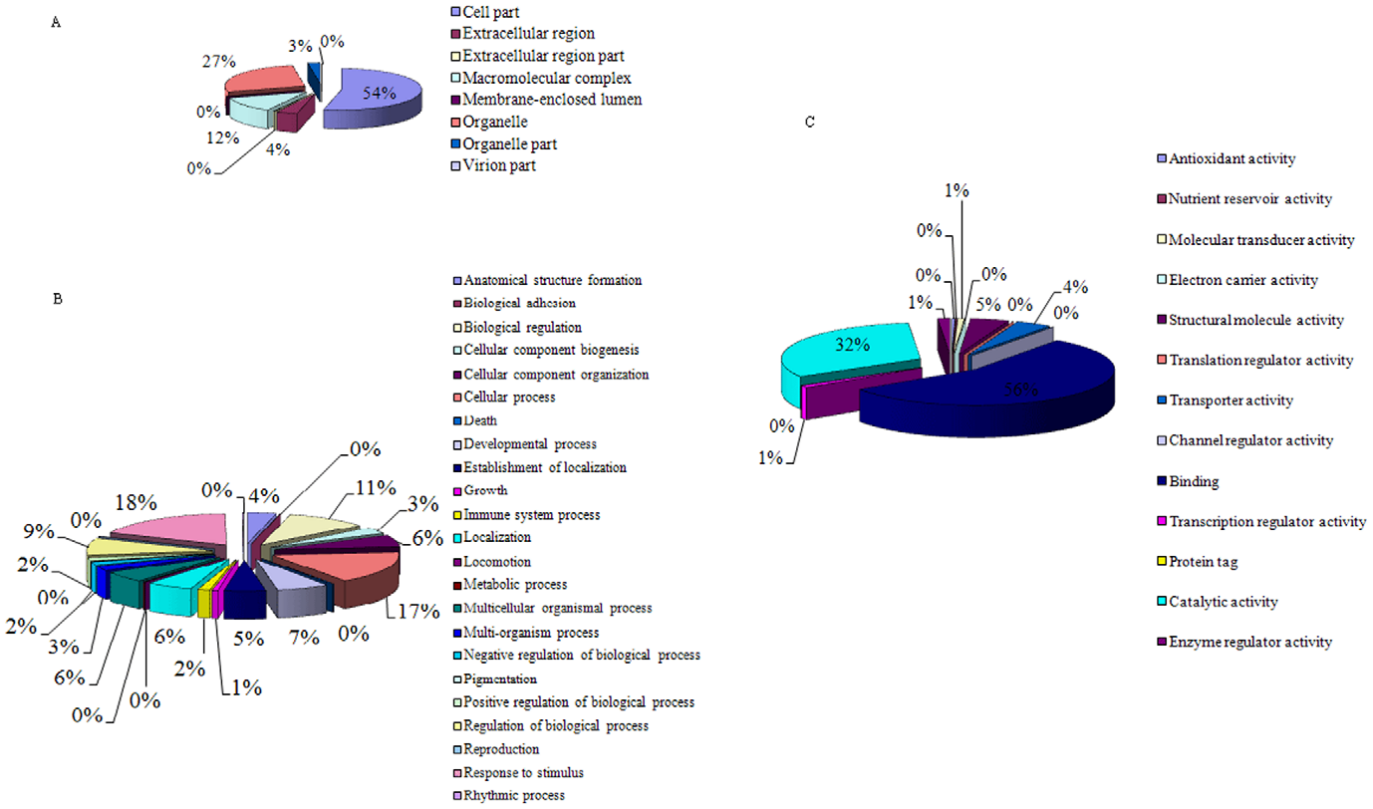
Gene Ontology (GO) analysis of 28,432 cotton annotated contigs. The three GO categories are presented: cellular component (A), biological process (B), and molecular function (C).

The 28,432 assembled cotton contigs were further annotated by BLASTx against the GO protein database, using an E-value cutoff of 1e-20, with 22,400 cotton ESTs finding a protein homolog (Figure 2). A total of 372 unique cellular component classes were identified for 13,657 ESTs (Figure 3A). According to annotation classification of GO database, the largest cellular component found for cotton ESTs was from cell part (6,810 contigs, 55%) and the smallest was from virion part (7 sequences, ∼0%). We infer that ESTs associated with the virion part could result from contamination by virus mRNAs. A total of 13,964 ESTs were associated with 1,628 GO categories for biological processes. The majority of biological processes identified are involved in responses to stimuli (18%) and cellular process (17%) (Figure 3B). Furthermore, 15,378 ESTs were classified as involved in 1,407 molecular functions. The major molecular functions were associated with binding (57%) and catalytic (32%) activities (Figure 3C). Based on KEEG annotations from GO proteins, we made pathway enrichment analysis for cotton ESTs. This revealed 3,176 contigs to be involved in 271 different pathways (File S1).

**Figure 4:**
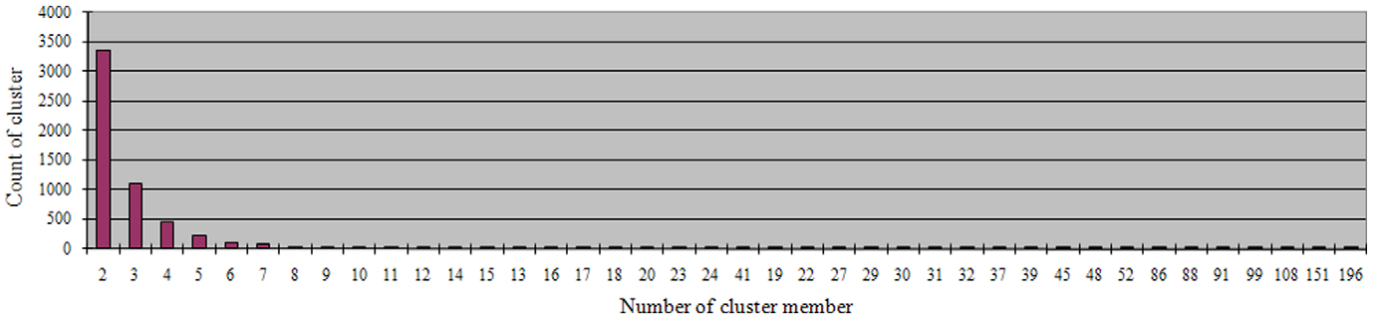
Cluster size distribution of cotton contigs.

Using BLASTn cutoffs for E-value (≤1e-30) and sequence identity (≥90%), a total of 5,461 gene clusters were identified from the entire set of 28,432 assembled cotton ESTs. The sizes of clusters varied from two to 196 members with an average size of 3.62 (Figure 4). The majority of clusters (3,358/59.8%) had 2 members.

### Genomic comparisons with other model plants

**Figure 5:**
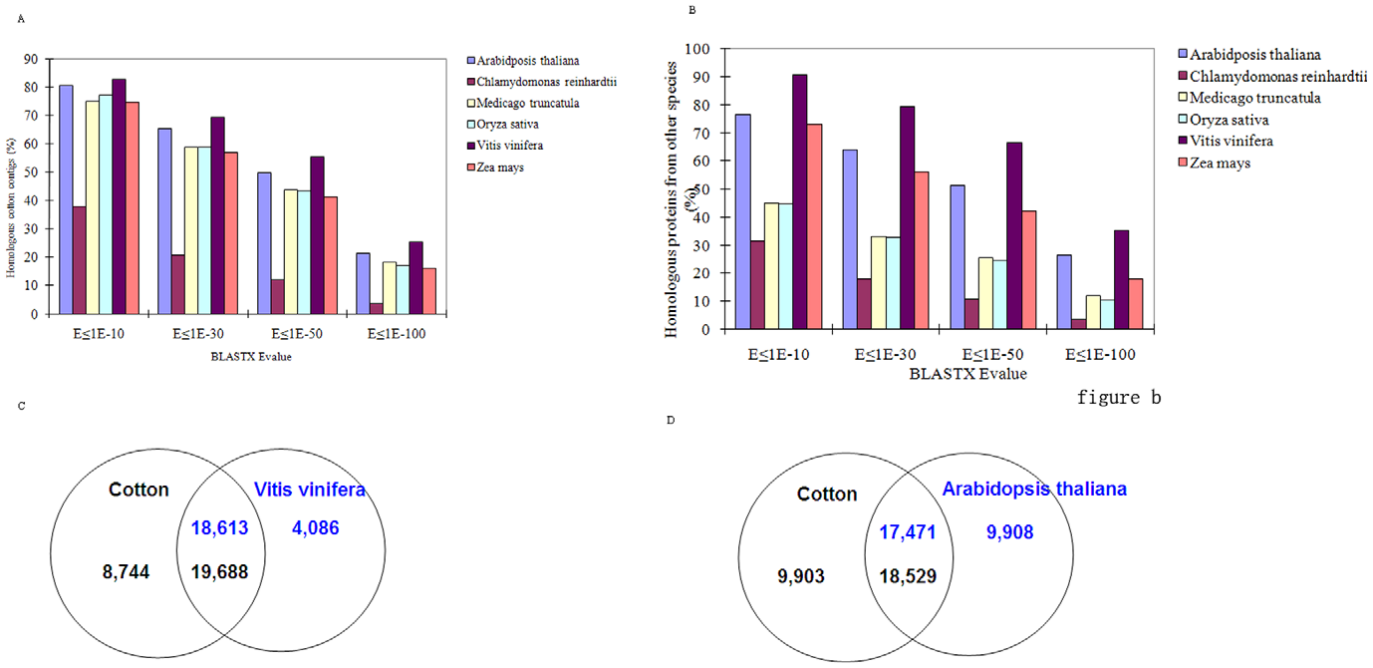
Homologous genomic comparison using several blast E-value cutoffs. A. Distribution of percent cotton contigs finding a hit in each genome. B. Distribution of cotton homologous proteins identified in other plant species. C. Comparison of number of homologs identified between cotton and *Vitis vinifera* with a BLASTx E-value cutoff of 1e-30. D. The same comparison between cotton and *Arabidopsis thaliana*.

Based on comparisons with reference protein databases from six model species, *Arabidopsis thaliana*, *Chlamydomonas reinhardtii*, *Medicago truncatula*, *Oryza sativa*, *Vitis vinifera*, *Zea mays*, cotton contigs were shown to be the most similar overall to *Vitis*, followed by *Arabidposis* (Figure 5); like cotton, both of these species are dicots. Using a BLASTx E-value cutoff of 1e-30, 18,613 of 22,699 (82.0%) sequences from *Vitis* were found to be homologous with 19,688 of 28,432 (69.2%) cotton ESTs (Figure 5C), whereas 17,471 of 26,379 (66.2%) sequences from *Arabidposis* were similar to 18,529 of 28,432 (65.1%) cotton contigs (Figure 5D). Amongst the six model species, *Chlamydomonas* was identified as having the least overall similarity (31.4%) to cotton. These data generally agree with current views of plant evolution; however, the highest overall similarity of cotton sequences to *Vitis* is somewhat surprising. Molecular phylogenetic analyses place the Malvaceae (cotton) and Brassicaceae (*Arabidopsis*) as sister families, with the Vitaceae (*Vitis*) a more distant outgroup [25]. The greater similarity between cotton and *Vitis* suggests that they retain somewhat more similar genome contents and sequence conservation from the common ancestor of all three taxa, than does *Arabidopsis*.

### miRNAs and their targets in cotton

Because of the limited nucleotide sequence resources available, miRNA-related research in cotton has lagged far behind other plant species. Currently, only 34 cotton miRNAs have been identified and deposited into the miRBase database [26]. In this study, we used a total of 2,454 known plant miRNAs deposited in miRBase (Release 15) [26] as a reference set, and identified 87 miRNAs among cotton EST contigs and raw ESTs (Table 3). Of these, 59 were identified for the first time in cotton.

**Table 3:** 87 miRNAs identified in cotton ESTs.

miRNA	Family	Mature sequence	LM*	Strand	Location	GC%	MFE	MFEI	EST Id	Data Type#
ghr-miR156d	156	UGACAGAAGAGAGUGAGCAC	20	−	5′	51.81	54	1.26	contig21398	Predicted
ghr-miR156e	156	UGAAGAAAGACAGAGCAU	18	−	5′	39.14	94.3	0.58	contig18605	Predicted
ghr-miR156f	156	UGAAGAAGAAAGAGAGCAU	19	+	5′	36.62	24.9	0.96	EV488115	Predicted
ghr-miR156g	156	UGAAGAAGAAAGAGAGAAG	19	+	3′	33.8	16	0.67	DW508826	Predicted
ghr-miR156h	156	UGAAGAAUAGAGCGAUCAC	19	+	3′	51.28	121.63	0.55	EV491219	Predicted
ghr-miR156i	156	UGAAGACCAGAGUGAGCAC	19	−	5′	41.47	79.5	0.64	AJ513999	Predicted
ghr-miR159	159	UUUGGAUUGGAGGGAGCUCUA	21	+	3′	47.02	72.7	0.92	ES824206	Predicted
ghr-miR162a	162	UCGAUAAACCUCUGCAUCCAG	21	+	3′	42.86	35.4	0.91	DW493971	Predicted
ghr-miR164	164	UGGAGAAGCAGGGCACGUGCA	21	−	5′	50.77	38.3	1.16	DR461140	Validated
ghr-miR164b	164	UGGAGAACAUGGGCACAUGGU	21	+	5′	37.52	138.1	0.72	contig25636	Predicted
ghr-miR164d	164	UGGAAAGCGGGCAGUGAG	18	−	3′	56.26	174.4	0.66	AJ514172	Predicted
ghr-miR166b	166	UCGGACCAGGCUUCAUUCCCC	21	+	3′	43.54	61.49	0.96	DW502146	Predicted
ghr-miR169	169	AAGCCAAGAAUGAAUUGCCUG	21	−	5′	51.47	65.5	0.62	DW509134	Predicted
ghr-miR171	171	AGAUUGAGCCGCGCCAAUAUC	21	+	3′	43.53	37.8	1.02	DW507416	Predicted
ghr-miR172	172	AGAAUCCUGAUGAUGCUGCAG	21	+	3′	34.74	38.21	1.16	ES839084	Validated
ghr-miR390a,c	390	AAGCUCAGGAGGGAUAGCGCC	21	+	3′	42.86	40.2	0.96	contig17644	Predicted
ghr-miR393	393	UCCAAAGGGAUCGCAUUGAUCU	22	+	5′	38.66	45	0.98	ES827656	Validated
ghr-miR394a	394	UUGGCAUUCUGUCCACCUCC	20	+	5′	48.19	35	0.88	ES802173	Validated
ghr-miR394b	394	UUGGCAUUCUGUCCACCUCC	20	+	5′	40.21	28.52	0.73	DW517361	Validated
ghr-miR395	395	CUGAAGUGUUUGGGGGAACUC	21	+	3′	52.94	55	1.02	DW501342	Predicted
ghr-miR396a,b	396	UUCCACAGCUUUCUUGAACUG	21	+	5′	40	43.3	0.94	contig21626	Predicted
ghr-miR398	398	UGUGUUCUCAGGUCACCCCUU	21	+	3′	50.75	32.1	0.94	DW498056	Validated
ghr-miR398b	398	UGUUUAUCAGGCACCCCUU	19	+	5′	49.15	12	0.41	contig28115	Predicted
ghr-miR399c	399	UGCCAAAGGAGAGUUGGCCUU	21	+	3′	47.3	31.7	0.91	DW510913	Validated
ghr-miR399d	399	UGCCAAAGGAGAUUUGCCCUG	21	+	3′	41.56	39.1	1.22	DW509341	Validated
ghr-miR399e	399	UGCCAAAGGUGCUGCUCUU	19	−	3′	57.35	28	0.72	contig21507	Predicted
ghr-miR408	408	UGCUCGCCUCAUCCUCUCU	19	+	5′	43.84	115.99	0.65	DR454452	Predicted
ghr-miR413	413	CUGGUUUCACUUGCUCUGAAC	21	+	3′	43.38	45.52	0.77	DW504189	Predicted
ghr-miR414a	414	GCAUCUUCAUCUUCAUCUUCA	21	+	3′	37.43	183.79	0.59	contig20173	Predicted
ghr-miR414b	414	UCAUCUUCUUCAUCAUCUUCG	21	−	5′	49.63	97	0.72	contig17531	Predicted
ghr-miR414c	414	UCAUCAUCAUCAUCACCUUCA	21	+	3′	46.51	29.9	0.75	contig20222	Predicted
ghr-miR414d	414	CCAUCUUCAUCAUCAUCAUCA	21	−	5′	48.82	76.7	0.62	ES799840	Predicted
ghr-miR414e	414	UCUCCUUCAUCAUCAUCGUCA	21	−	3′	44.33	14.7	0.34	DW502456	Predicted
ghr-miR414f	414	UCAUUUUCAUCAUCAUCGUCA	21	−	5′	42.74	48.85	0.47	ES835113	Predicted
ghr-miR414g	444	UGCAGUUGUUGUCUAUGCCU	20	−	5′	42.64	32.1	0.58	AJ513351	Predicted
ghr-miR479	479	CGUGAUAUUGGUUCGGCUCAUC	22	+	5′	37.88	32.6	1.3	ES809290	Validated
ghr-miR482a	482	UCUUUCCUACUCCUCCCAUACC	22	+	3′	40	33.5	0.99	DR457519	Validated
ghr-miR482b	482	UCUUGCCUACUCCACCCAUGCC	22	+	3′	46.94	43.9	0.95	DT527030	Validated
ghr-miR482c	482	CCUCCUCCUCUCCAUUGC	18	+	3′	50.26	70.7	0.72	ES808713	Predicted
ghr-miR482d	482	UCUUCUUCUUCCUCCCAUC	19	−	3′	52.44	32.7	0.76	DT464811	Predicted
ghr-miR528	528	UGGAAGGGNGCAUGCAUGGAG	21	+	3′	34.41	43.7	0.68	DN804697	Predicted
ghr-miR529a	529	AGAAGGAGAGAGUCAACUU	19	+	3′	39.22	11.8	0.59	contig4544	Predicted
ghr-miR529b	529	UUUUCCCCUCUCUCUUCUUC	20	+	5′	42.06	33.86	0.64	contig26549	Predicted
ghr-miR529c	529	CUGUACUCGCUCUCUUCAUC	20	−	3′	48.44	114.3	0.61	DT046423	Predicted
ghr-miR530	530	UGCAUUUGCAAUCUGCUCCUA	21	+	3′	41.27	20.9	0.8	contig16357	Predicted
ghr-miR808	808	AUGAAUGUGGGAAAUGCUAGAA	22	−	3′	29.79	56.9	2.03	EX172412	Predicted
ghr-miR827a,b,c	827	UUAGAUGACCAUCAACAAACA	21	+	3′	37.4	39.2	0.85	contig22556	Validated
ghr-miR835	835	UUCUUCAUUGUUCUUUCUC	19	+	5′	36.78	57.94	0.6	DW506095	Predicted
ghr-miR838a	838	UUUUCUUCUCCUUCUUUACA	20	+	3′	42.7	27.2	0.72	DW516621	Predicted
ghr-miR838b	838	UUUUCUUCUACUUCUAGCAUU	21	−	5′	44.26	54.4	0.67	DW476363	Predicted
ghr-miR847a	847	UCACUCCUUUCCUUGAUG	18	−	3′	32.94	17.5	0.63	contig27404	Predicted
ghr-miR847b	847	UCACUCUCUUCUUUUGUUG	19	−	3′	36.21	13.65	0.65	contig23150	Predicted
ghr-miR855	855	AGGAAAAGAAAGGAAAAGGAA	21	−	3′	42.76	118.7	0.64	CO499070	Predicted
ghr-miR1132a	1132	GAUUAGGGACGGAAGGAG	18	+	5′	47.26	69.4	0.73	contig11460	Predicted
ghr-miR1132b	1132	CAUUAUGGCCAGAAGGAG	18	−	5′	49.8	85.4	0.67	contig26869	Predicted
ghr-miR1134	1134	UAACAACAACAAGAAGAAGGAGCU	24	+	5′	40.63	46.8	0.6	contig18889	Predicted
ghr-miR1144	1144	UGGAACCGUGGCAGGAGGAG	20	−	3′	62.96	76.6	0.75	contig5195	Predicted
ghr-miR1161	1161	UACUGGAGUUCUCAAGAAA	19	−	3′	32.73	14.6	0.81	DV849247	Predicted
ghr-miR1444	1444	UCCACAUUGGGUAAUGGUC	19	+	3′	33.67	68.1	1.03	contig21923	Predicted
ghr-miR1507	1507	UCUCUUCCAUGCAUCUUCUGA	21	−	3′	40.45	28.5	0.79	DT048287	Predicted
ghr-miR1509	1509	UUAAUGUAAAAAUACGGUG	19	−	3′	22.67	8.4	0.49	contig12637	Predicted
ghr-miR1533a	1533	AUAAUAAAAAGAAAAGGA	18	+	5′	27.05	25.6	0.78	contig21520	Predicted
ghr-miR1533b	1533	CUAAUAAUAAUAAUAAUGU	19	+	3′	20.69	5.87	0.49	contig15142	Predicted
ghr-miR1533c	1533	AGAUUAAAAAUAAUAAUGU	19	+	3′	30.3	11.9	0.6	DR453981	Predicted
ghr-miR1533d	1533	AAAAUAAAAAUAAAAGGA	18	+	3′	10.61	6.36	0.91	DT561626	Predicted
ghr-miR1533e	1533	AUAAUUAAAAAUAAUAAUUU	20	+	5′	28.11	53.4	0.68	AI055426	Predicted
ghr-miR1533f	1533	AAAUUAAAAAUAAUAAUAA	19	−	3′	34.23	45.41	0.89	CD486467	Predicted
ghr-miR1535a	1535	CGUUUUUGUGGUGAUGGUCU	20	−	3′	41.92	121.4	0.63	contig21820	Predicted
ghr-miR1535b	1535	CUUGUUUGUGAUGUGUGU	18	−	5′	36.62	148.8	0.72	contig21907	Predicted
ghr-miR1854	1854	UGGGCCAUUUGUAGAUUGGA	20	+	5′	32.73	11.36	0.63	DT459810	Predicted
ghr-miR1857	1857	UGGUUUUUCUUGGAGAUGAAG	21	+	3′	41.64	83.44	0.68	ES792140	Predicted
ghr-miR1860	1860	AUCUGAGAAGCUAGGUUUUCUUU	23	+	3′	28.28	37.8	0.68	DW494072	Predicted
ghr-miR1862	1862	ACAAGGUUGGUAUAUUUUAGGACG	24	+	3′	40.32	22.6	0.9	EX172412	Predicted
ghr-miR1869	1869	UGAGAACAAUAGGAUGGGAGAUA	23	−	3′	39.19	18.86	0.65	contig14048	Predicted
ghr-miR1884	1884	AAUGUAUGACGCUGUUGACUUUUC	24	+	5′	23.83	45.2	0.98	EX172380	Predicted
ghr-miR2529	2592	AAAUCUUGAAUCAUGUGUU	19	−	3′	44.82	184.51	0.47	contig14636	Predicted
ghr-miR2595	2595	UCCAUUUUCUUCUUUCUUCU	20	+	5′	39.04	94.12	0.72	contig19425	Predicted
ghr-miR2635	2635	AUUAUUGUCAAGUGUCUUG	19	+	5′	25.76	8.45	0.5	contig4047	Predicted
ghr-miR2645	2645	UUUAUAGAAUGAGCAUAUAC	20	−	3′	30.97	25.6	0.73	AJ513108	Predicted
ghr-miR2673	2673	CCUCUUCCUCUUCCUCUUCUUC	22	−	5′	38.99	69.6	0.47	ES825617	Predicted
ghr-miR2868	2868	UUGAUUUUGGUAGAAGAAA	19	+	5′	35.19	24	0.63	contig17454	Predicted
ghr-miR2876	2876	UUCCUCUAUGGACACUGUUUC	21	+	5′	42.03	177.72	0.58	contig24591	Predicted
ghr-miR2938	2938	GAGCUUUGAGAGGGUUCCGG	20	−	3′	52.33	26.6	0.59	CD485951	Predicted
ghr-miR2948-5p	2948	UGUGGGAGAGUUGGGCAAGAAU	22	+	5′	45.83	30.9	0.94	DW517596	Validated
ghr-miR2949a,b,c	2949	UCUUUUGAACUGGAUUUGCCGA	22	+	5′	43.04	27.3	0.8	contig9309	Validated
ghr-miR2950	2950	UGGUGUGCAGGGGGUGGAAUA	21	+	3′	49.35	43.1	1.13	DW514754	Validated
ghr-miR3476	3476	UGAACUGGGUUUGUUGGCUGC	21	+	5′	37.23	38	1.09	DW497660	Validated

*Length of mature miRNA sequence.

# Validated means that the miRNA was confirmed by experimental methods (deep sequencing, qRT-PCR or direct cloning).

**Figure 6:**
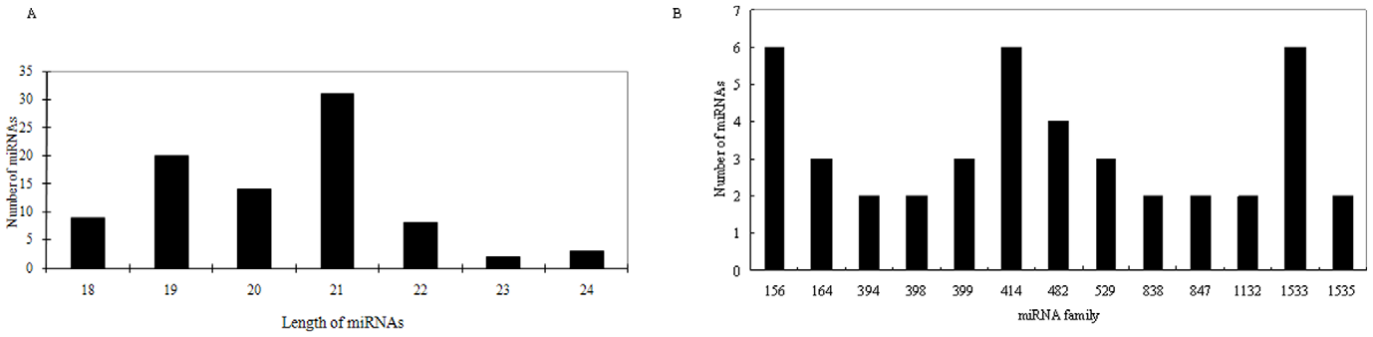
A. Distribution of length of miRNAs in cotton. B. Size distribution of cotton miRNA families with more than one member.

Of the 87 miRNAs identified, 33 were from our newly assembled contigs and 54 came directly from raw EST reads (Table 3). The length of the cotton miRNAs varied from 18 to 24 nt, with average of 20.3±1.4 nt (Figure 6A). The most abundant cotton miRNAs were 21 nt in length. These results are similar to miRNA lengths reported previously in plants [27]. The 87 miRNAs from cotton clustered into 57 families. The size of miRNA families in cotton varied from one to six sequence members (Table 3); 44 of 57 (77.2%) families had only one member (e.g., miR159, miR162, miR166, miR171, miR172, miR390, miR393, and miR395), whereas 13 (22.8%) had multiple members (e.g., miR156, miR164, miR394, miR398, miR399, miR414, and miR482) (Figure 6B). The largest miRNA families, including miRNA156, miRNA414, and miRNA1533, each with six members. Thirty-two of 87 miRNAs in cotton were obtained from the antisense strand of our original contig or EST, and the other 55 came from the sense strand (Table 3). miRNAs are located at either the 5′ or 3′ end of the hairpin arm. Our results show 50 of 87 miRNAs to be located at the 3′ end and 37 at the 5′ end.

Given that miRNAs target the transcripts of protein-encoding genes, a total of 18,621 ESTs, with E-values of less than 1e-25 in BLASTx searches against the plant protein database, were selected as a subject dataset for target prediction. Based on a discrete set of criteria (see experimental procedures), 87 miRNAs identified in cotton were found to target a total of 3,260 protein-encoding genes (File S2). Our target prediction suggests that cotton miRNAs regulate the expression of many types of genes associated with diverse biological and metabolic processes, including metabolic pathways, hormone signal transduction, stress response, and fiber development. As in previous investigations, validated miRNA-target pairs also were identified in cotton, including miR156-squamosa promoter-binding protein (SBP) [28], miR164-NAC domain protein (NAC) [29], miR398- Cu/Zn superoxide dismutase [30], miR172-AP2 domain-containing transcription factor [31], and miR393-transport inhibitor response 1 [28]. In addition, because cotton is one of most important fiber crops, we also carefully examined targets associated with fiber development or fiber yield. Amongst the potential miRNA targets identified in cotton, there were at least 23 genes tightly associated with fiber development (Table 4). These targets control cellulose synthesis (miR156g and contig16368), fiber development (miR414b and contig7645), and glucose metabolism (miR529a and contig16806).

**Table 4:** Potential targets of cotton miRNAs associated with fiber development.

MiRNA	Family	Target	Function	Type
ghr-miR156g	156	contig16368	Cellulose synthase	Fiber development
ghr-miR156g	156	contig18138	Glycosyl transferase, CAZy family GT43	Fiber development
ghr-miR156g	156	contig4371	Glycosyltransferase QUASIMODO1	Fiber development
ghr-miR156f	156	contig13757	Glycosyltransferase, CAZy family GT8	Fiber development
ghr-miR156g	156	contig17691	Glycosyltransferase, CAZy family GT8	Fiber development
ghr-miR156f	156	contig8831	Sugar transporter	Fiber development
ghr-miR156f	156	contig1543	UDP-glucuronate 5-epimerase	Fiber development
ghr-miR414b	414	contig7645	Similar to fiber protein Fb2	Fiber development
ghr-miR414e	414	contig22187	Sugar transporter	Fiber development
ghr-miR529a	529	contig16806	Glycosyl hydrolase family 17 protein	Fiber development
ghr-miR529b	529	contig23483	Glycosyl hydrolase family 17 protein	Fiber development
ghr-miR529a	529	contig19551	Glycosyltransferase, CAZy family GT8	Fiber development
ghr-miR529b	529	contig8845	Sugar transporter, putative	Fiber development
ghr-miR1533e	1533	contig22176	Glycosyltransferase, CAZy family GT47	Fiber development
ghr-miR1533e	1533	contig9681	UDP-glucose 4-epimerase	Fiber development
ghr-miR1533d	1533	contig20591	UGT73C6 (UDP-glucosyl transferase 73C6)s	Fiber development
ghr-miR1533d	1533	contig2536	Xyloglucan endotransglucosylase/hydrolase protein 22 precursor	Fiber development
ghr-miR1533b	1533	contig71	Xyloglucan endotransglucosylase/hydrolase protein 9 precursor	Fiber development
ghr-miR1535b	1535	contig21984	Sucrose synthase	Fiber development
ghr-miR2595	2595	contig8413	Glycosyl transferase family 2 protein	Fiber development
ghr-miR2595	2595	contig9765	Sugar transporter	Fiber development
ghr-miR2595	2595	contig24807	Xylulose kinase	Fiber development
ghr-miR2635	2635	contig2406	Xylose isomerase	Fiber development

### Sequence polymorphisms

We detected a total of 149,614 putative SNPs in 14,516 cotton contigs and 27,956 putative insertions/deletions (indels) in 8,674 contigs. Both SNPs and indels were detected in a total of 8,118 contigs. Our results show that SNPs occur once every 215 nt in cotton ESTs and indels occur once every 1,111 nt. The maximum frequencies of SNP and indels were 0.122 and 0.069 respectively. We generated a standard normal distribution to analyze the frequencies of SNPs/indels among contigs, and determine which contigs had a significantly high number of SNPs at P<0.05 (significant) and P<0.01 (highly significant). We found 1,933 contigs to contain significant SNP frequencies, with 802 of these contigs at high significance. A significant frequency of indels was found for 1,089 contigs, 735 of which were highly significant. Currently, the genome of cotton is incompletely sequenced; in its absence, however, the large resource of ESTs available allow for identification of large numbers of SNPs [14]. The apparently high frequency of SNPs and indels we observed in cotton ESTs could be due in part to sequencing errors. To address this issue, we followed the criteria of Wang and co-workers [14] to remove pseudo-SNPs and pseudo-indels as much as possible. Without experimental validation, however, it is difficult to determine whether a given SNP or an indel in cotton represents a real polymorphism. Nevertheless, we suggest that the high average frequency of SNPs we observed could, indeed, reflect real genetic variation resulting from the complicated genetic background present in large cotton EST libraries. However, because of the nature of cotton EST data in the NCBI database, it is not 100% sure that these SNPs are really SNPs or caused by sequencing errors. As deep sequencing technology become available, more study may be performed to investigate this issue.

Aside from those that could not be assigned a presumed function, many cotton EST contigs with significant rate of SNPs and indels are associated with transcription factors, energy metabolism, stress response, signal transduction, and protein kinases (File S3). A previous investigation showed that high SNP frequency (0.013) occurred in R2R3-MYB transcription factors from cotton [32]. In this study, we also detected two contigs (contig2733 and contig15263) annotated to encode MYB transcription factors that have significantly high SNP frequencies. Therefore, it is possible that the high diversity of SNPs and indels in the cotton transcriptome could be related to functional adaptations to environmental stress.

### Simple sequence repeats

Because of their relative abundance and ease of generation, SSRs are among the most powerful of molecular markers, and have been applied widely in molecular-assisted selection (MAS) for plant breeding programs [33]. SSR markers derived from expressed sequence tags (EST-SSRs) originate from transcribed regions of the genome and are likely to be even more transferable across lines, populations and species than random genomic SSRs [13]. In this study, we analyzed SSRs in both cotton contigs and raw ESTs. We identified a total of 151 SSRs from cotton contigs and 4,214 from raw ESTs (File S4). Among SSRs from contigs, the most abundant repeat types were trinucleotides (130, 86.09%) followed by dinucleotides (21, 13.91%). The dominant sequence repeat in contigs was AAG/CTT (10, 6.62%) followed by TGA/TCA (9, 5.96%). Trinucleotide repeats also were the most common among SSRs from raw ESTs (2,961, 70.27%) again followed by dinucleotides (829, 19.67%) along with a sizeable fraction of tetranucleotides (424, 10.06%). Dominant repeat types in raw ESTs were GAA/TTC (159, 3.77%) and GAT/ATC (159, 3.77%). Amongst the 151 SSR markers found, only 43 come from the contigs annotated with known functions. Potentially, these markers could be exploited for use in marker-assist breeding selection. Of these SSRs, 51 from contigs and 1,663 from raw ESTs have not been reported previously in cotton.

In further investigate the potential of these SSR repeats as genetic markers, we employed eprimer3 (primer 3) to design primer pairs for each SSR under a series of primer-designing parameters (see Experimental procedures). We were able to find viable primer pairs for 121 of 151 contig SSRs and 3,092 of 4,214 raw EST SSRs (all these primers can be downloaded from the cotton EST website www.leonxie.com).

### Web-based database for cotton ESTs

**Figure 7:**
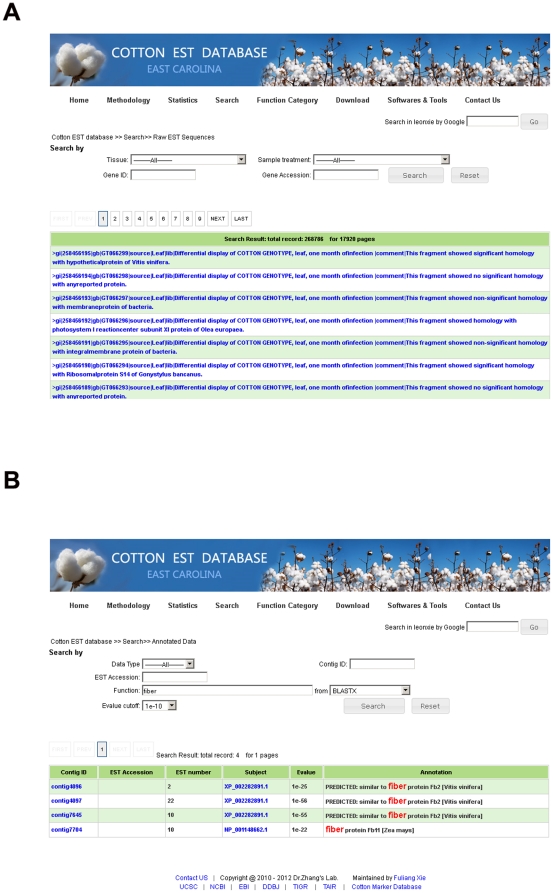
Interface of cotton EST database for querying raw ESTs (A), and assembled contigs (B).

To facilitate further investigation and application of cotton genome-related research, we constructed a web-based, searchable and downloadable database for managing cotton ESTs data, along with related deep sequence analyses including assembly, annotation, miRNAs, SNP and indels, and SSRs (Figure 2). This database can be accessed freely through a web interface (www.leonxie.com). Raw ESTs, as well as annotation and assembly data can be queried using different strategies, such as gene accession, gene ID, and function (Figure 7). We also incorporated the Cotton Marker Database (CMD) into our web-server and built connections with raw EST, assembled contigs, and SSR databases. In this way, users can quickly access marker information from cotton ESTs or access marker-related ESTs through CMD markers. We have attempted to develop a seamless connection among all of these cotton EST datasets and resources. For instance, when investigating a contig, users can visit its related information, including functional annotation, miRNA, SSR, SNP, GO, and KEGG; alternatively that contig can be accessed from any one of the related resources as a starting point. To improve the efficiency of BLAST analyses of cotton ESTs, we also built a local WWW-BLAST server permitting directed and advanced BLAST options. Raw cotton ESTs, assembled contigs, consensus assemblies, singletons, all reference protein databases from plants, and all reference plant nucleotide databases are incorporated within our local WWW-BLAST server as potential query targets. Furthermore, EST data and related analytical tools and results, all can be freely accessed and downloaded.

### Conclusions

We have developed a specific and dedicated workbench for assembling cotton ESTs and for performing genome-wide analyses of the cotton transcriptome. In addition to raw ESTs and assembled contigs, additional EST-related information, including miRNAs, SNPs, and SSRs has been integrated into this database. A friendly web-interface allows users to access and download these data as batch files or via directed searches based on specific interests and needs. Moreover, now that this platform for cotton EST data has been established, it will be very convenient to add new cotton ESTs and annotated resources to our database in future. Therefore, this cotton EST database can contribute significantly to advancing research on cotton ESTs and global genome-wide analyses.

## Methods

### Dataset

A total of 268,786 cotton ESTs (*Gossypium hirsutum L.*) were downloaded from NCBI (http://www.ncbi.nlm.nih.gov/). These ESTs were obtained from at least 90 EST libraries and samples treated under at least eight different abiotic and biotic conditions.

### Data pre-processing

A majority of raw EST sequences potentially contain various contaminating elements, such as sequencing primers, vector sequence, sequences from other species, and sequencing errors. In addition, poly A/T tail and low complexity sequences are inevitably present in some raw ESTs. Thus, a critical first step is to remove these contaminated sequences before performing more deep analysis. In this study, we first cleaned original cotton ESTs by Seqclean [34] (ftp://ftp.tigr.org/pub/software/tgi/seqclean/) from TIGR under default parameters. Seqclean is a versatile tool for removing sequences from vectors, mitochondria, ribosomal RNAs, sequencing primers, polyA/T tails, low complexity sequences, and sequences with lengths under 100 nt [34]. After processing with SeqClean (Figure 2), we employed RepeatMasker (version 3.2.9, http://www.repeatmasker.org/) to mask repeated elements based on Repbase (Repbase 15.04, http://www.girinst.org/) [35]. Finally, a total of 235,328 cleaned ESTs were kept for further assembly.

### EST clustering and assembling

The cleaned EST sequences were clustered and assembled into contigs (consensus and singletons) by TGICL (ftp://ftp.tigr.org/pub/software/tgi/tgicl/) [36], which could partition the input dataset into small groups of sequences (clusters) using Megablast and assemble each cluster by using the cap3 program [37] into contigs. The resulted data was further performed an ortholog search against the published assembled data of Gossypium's ESTs (http://www.agcol.arizona.edu/cgi-bin/pave/Cotton/index.cgi) [7] using Orthomcl (Version 2.0, http://orthomcl.org/cgi-bin/OrthoMclWeb.cgi?rm=orthomcl#Software) under the cutoff of E-value of 1e-25 and identify of 95%.

### Functional annotation

In order to investigate putative functions of cotton ESTs, we performed BLASTx [38] against reference protein databases from all plants using an E-value cutoff of 1e-20, and BLASTn against reference nucleotide acid databases from all plants at an E-value cutoff of 1e-25. Only the best high-scoring segment pair (HSP) was kept for annotation. We also tried to annotate possible open reading frames (ORFs) of contigs and further infer their protein sequences by GETORF from Emboss tools package (http://emboss.sourceforge.net/). The longest ORF was considered to be the candidate CDS sequence, and its translation the presumed protein sequence as well.

To better understand the functional classification of ESTs, contigs were used as queries in BLASTx using Gene Ontology (GO) analysis [39]. Cellular component, biological process, and molecular function were classified for these contigs. We performed further pathway enrichment according to GO annotations for Kyoto Encyclopedia of Genes and Genomes (KEGG) [40].

### Cluster analysis

Each individual contig was queried against the complete assembled EST data set using BLASTn. All contigs hit by the query with an E-value of less than 1e-30 and an identity of more than 90% were defined as a cluster.

### Overall genomic sequence similarity

Using different BLASTx E-value cutoffs (E≤1e-10, E≤1e-30, E≤1e-50, and E≤1e-100), we investigated sequence similarity between the cotton contigs we obtained and reference cDNA databases from several model species; these included *Arabidposis thaliana* (TAIR9, ftp://ftp.arabidopsis.org/Sequences/blast_datasets/TAIR9_blastsets/), *Chlamydomonas reinhardtii* (Chlre4, http://genome.jgi-psf.org/chlamy/chlamy.download.ftp.html), *Medicago truncatula* (Mt3.0 release, http://www.medicago.org/genome/downloads.php), *Vitis vinifera* (ftp://ftp.ncbi.nih.gov/genomes/Vitis_vinifera/Assembled_chromosomes/), *Zea mays* (http://www.plantgdb.org/ZmGDB/cgi-bin/downloadGDB.pl), and *Oryza Sativa* (version 6.1, ftp://ftp.plantbiology.msu.edu/pub/data/Eukaryotic_Projects/o_sativa/annotation_dbs/pseudomolecules/version_6.1/all.dir/).

### Sequence polymorphism analysis

Based on assembly results of consensus contigs, SNP and indel polymorphisms were analyzed. A perl script was developed to detect SNPs and indels under several criteria as described by Wang and co-workers [14]. Briefly, 1) a mismatch identified within contigs containing more than four individual EST reads was definable as a SNP or an indel; 2) variation among sequences was considered to be a bona fide SNP or indel polymorphism when it was found at least twice within contigs assembled by 5–6 ESTs; 3) at least three times within contigs assembled by 7–8 ESTs; 4) at least four times within contigs assembled by 9–12 ESTs; 5) and at least five times within contigs assembled by 13 or more ESTs.

### Identification of miRNAs and their targets

MicroRNAs (miRNAs) are known as a class of none-coding endogenous small RNA molecules with lengths of ∼21 nt. Investigations increasingly show that miRNAs regulate target mRNAs either by inducing their degradation or by inhibiting translation [20]. To date, miRNAs have been predicted successfully from various EST [41] and GSS databases [23]. Especially for those species without complete genome information, an EST database is considered to be an ideal data source for predicting miRNAs their targets as well [24], [42]. In our analysis, low complexity sequences, sequences with lengths of less than 100 nt, and sequences with repeated elements were removed in data pre-processing; EST contigs generated and raw ESTs then were combined as the subject dataset. We employed all known plant miRNAs from miRBase (Release 15: April 2010, http://www.mirbase.org/) [26] as a reference set and performed homology searches against the subject dataset using methods reported previously [43], [44]. Cotton miRNA targets also were predicted according to method in previous reports [43].

### SSR detection and primer design

In order to locate simple sequence repeats (SSRs) in cotton ESTs, we performed SSR analyses on cotton contigs and raw ESTs using a software SSR Finder from GRAMENE (ftp://ftp.gramene.org/pub/gramene/software/scripts/ssr.pl). The parameters were designed for identifying perfect di-, tri-, tetra-, penta-, and hexa-nucleotide motifs with a minimum of 6, 5, 4, 4, and 4 repeats respectively [9]. Eprimer3 from EMBOSS bioinformatics software packages (http://emboss.sourceforge.net/) [45] was used to design flanking primers for detected microsatellites. The major parameters for primer design were set as following: PCR products ranging from 100 to 300 nt; primer lengths ranging from 18 to 24 nt with an optimum of 20 nt, 60°C optimal annealing temperature, and GC content from 40%∼65% with an optimum of 50% [9].

### Construction of a web-based cotton EST database

In order to share our integrated data and analytical results on cotton ESTs, including raw ESTs, assembled EST contigs, predicted miRNAs, sequence polymorphisms, and SSRs and primers, we integrated the information from each step of our investigation into a web-based cotton EST database, using open-source software (Apache, PHP, and MySQL), and constructed interfaces among the data types (Figure 2). Furthermore, to facilitate access to potentially useful markers from cotton raw ESTs and assembled contigs, we incorporated current data (SSR and QTL) from the Cotton Marker Database (CMD) (http://www.cottonmarker.org/) into our EST database. Our new web-based cotton EST database provides users with a friendly interface to query or download data. It is freely available at the website www.leonxie.com.

## Supporting Information

File S1 **Pathway analysis by KEGG.**
(XLS)

File S2 **Predicted miRNA targets.**
(XLS)

File S3 **Cotton EST contigs with significant SNPs and indels.**
(XLS)

File S4 **Identified SSR markers with designed primers.**
(XLS)
